# Supplementary material for: A fluorescence anisotropy assay to discover and characterize ligands targeting the maytansine site of tubulin
Source: Nat Commun. 2018 May 29;9:2106. doi: 10.1038/s41467-018-04535-8 (PMC5974090; doi:10.1038/s41467-018-04535-8)
Supplement: Supplementary file 1 — Supplemental Information [file 41467_2018_4535_MOESM1_ESM.pdf]

SUPPLEMENTARY INFORMATION

A fluorescence anisotropy assay to discover and characterize ligands targeting the maytansine-site of tubulin

Menchon et al.

Supplementary Figures

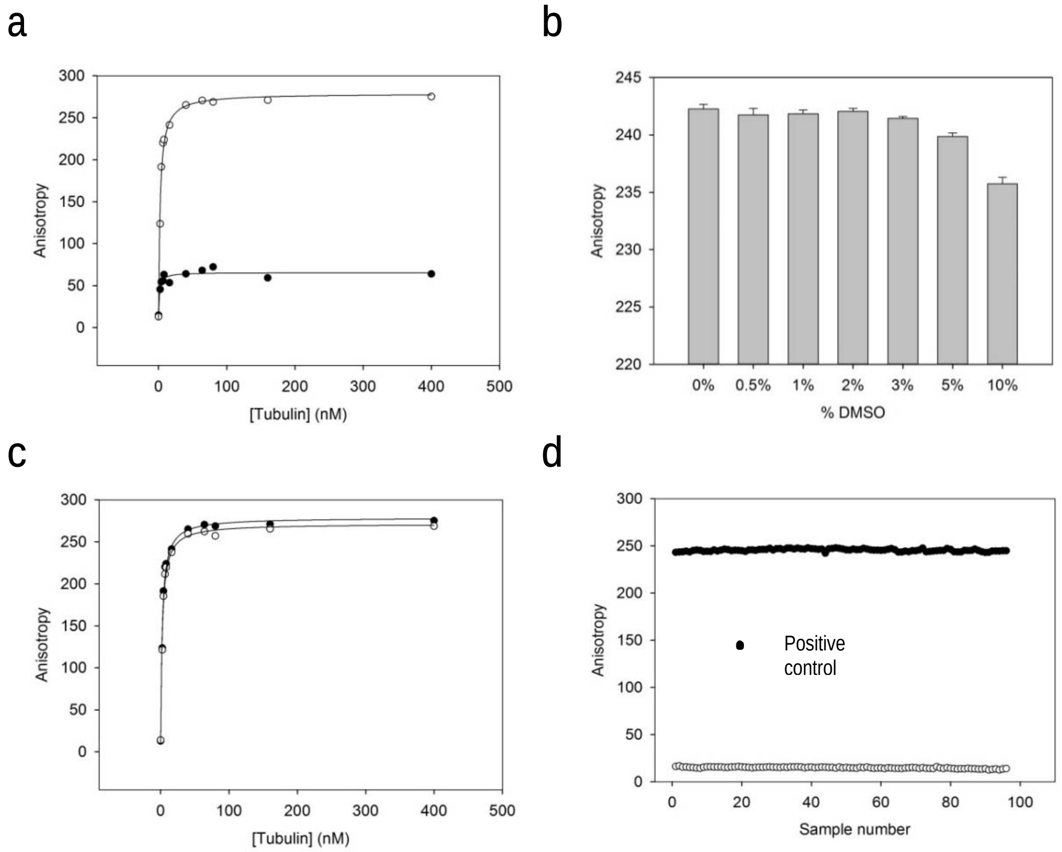

Supplementary Fig. 1: Characterization of the fluorescence polarization-based FcMaytansine assay.

a Anisotropy variation as a function of tubulin and initial FcMaytansine concentrations (closed circles, 1 nM FcMaytansine; open circles, 10 nM FcMaytansine).

b Anisotropy variation as a function of DMSO concentration. The data are from three independent experiments and represent mean ± SEM. The solid lines represent fits to the data.

c Anisotropy variation as a function of tubulin concentration and incubation time (closed circles, 15 min; open circles, 45 min).

d Analysis of the assay's Z-factor in a 96 well plate format. The closed circles are the positive (mixture of 400 nM tubulin + 10 nM FcMaytansine) controls and open circles are the negative (10 nM FcMaytansine in buffer alone) controls. The Z-factor is 0.97 and was calculated according to Zhang et al.[3]. It is a measure of the statistical effect size and reports on an assay's signal dynamic range and the data variation associated with the signal measurements. The Z-factor has been proposed for the use in high-throughput screening to judge whether the response in a particular assay is large enough to warrant further attention.

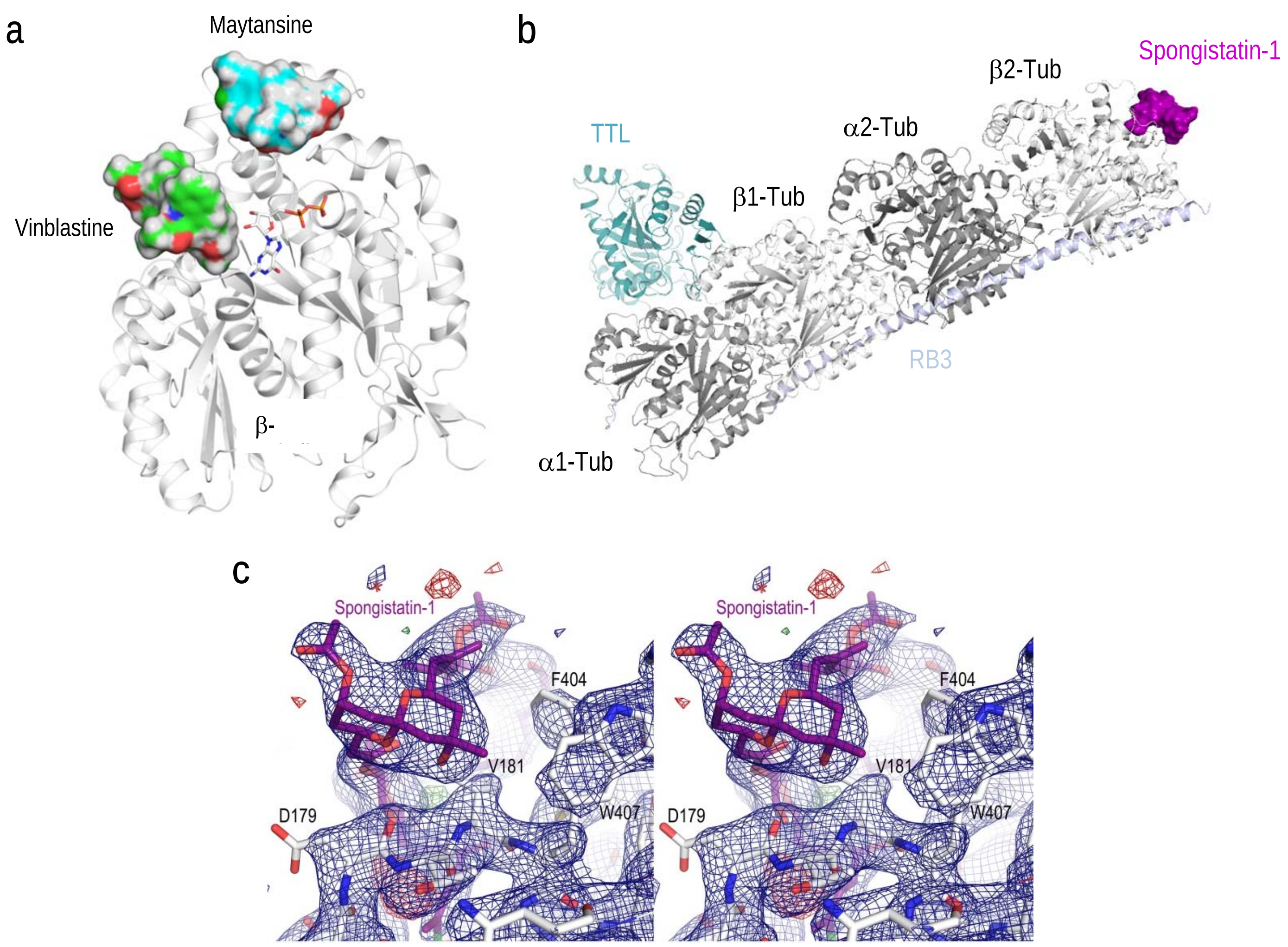

Supplementary Fig. 2: Binding of ligands to β-tubulin.

a Close-up view of the interaction of maytansine and vinblastine with tubulin. The β-tubulin subunit is represented in light gray cartoon representation. Maytansine and vinblastine are shown in cyan and green surface representation, respectively. The GDP molecule is shown in orange spheres representation.

b Overall view of the T_2R-TTL-spongistatin complex structure. Tubulin (dark and light grey), RB3 (light blue) and TTL (teal) are shown in ribbon representation. Spongistatin (purple) is in surface representation.

c Stereo image of a representative portions of the electron density maps of the T_2R-TTL-spongistatin-1 complex structures. The sigma A weighted 2mFo-DFc (dark blue) and mFo-DFc (green (+); red (-)) electron density maps are contoured at 1.0 and +/- 3.0 sigma, respectively. The protein is in white stick representation, the ligand is colored according to the same color code as in Figure 4e. Water molecules are represented in red spheres.

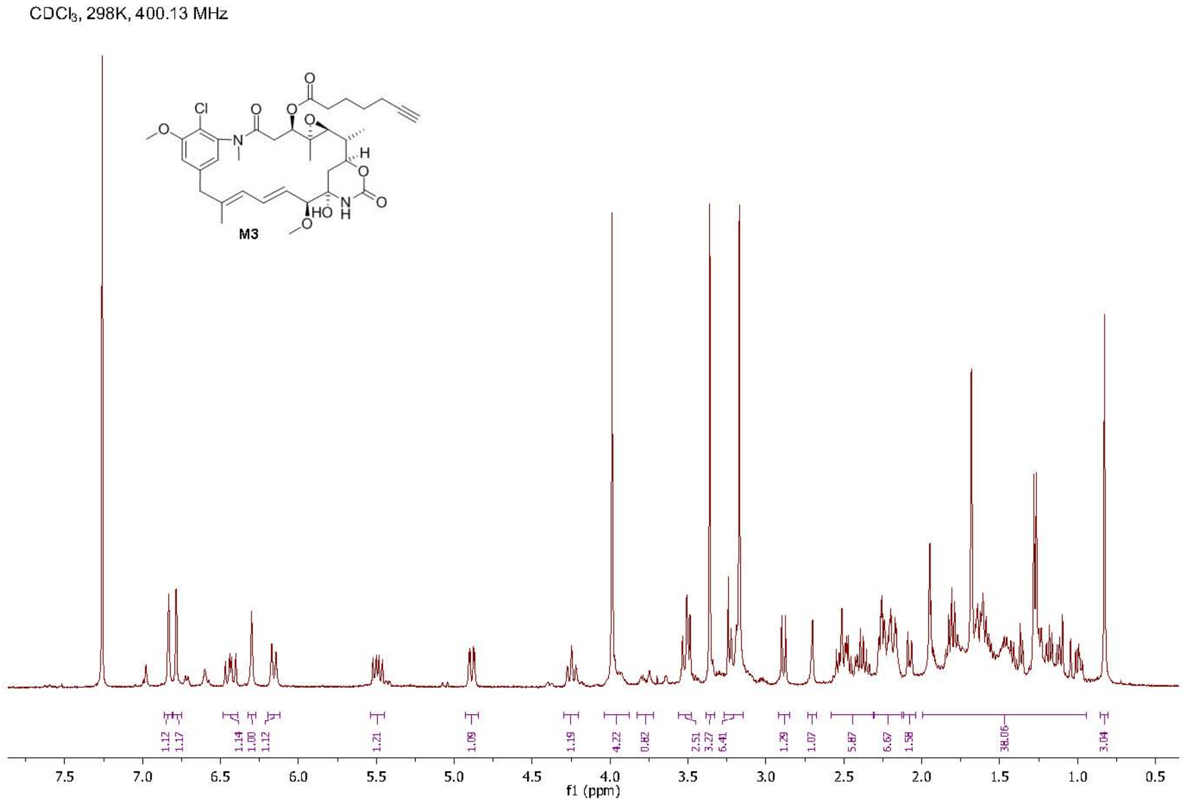

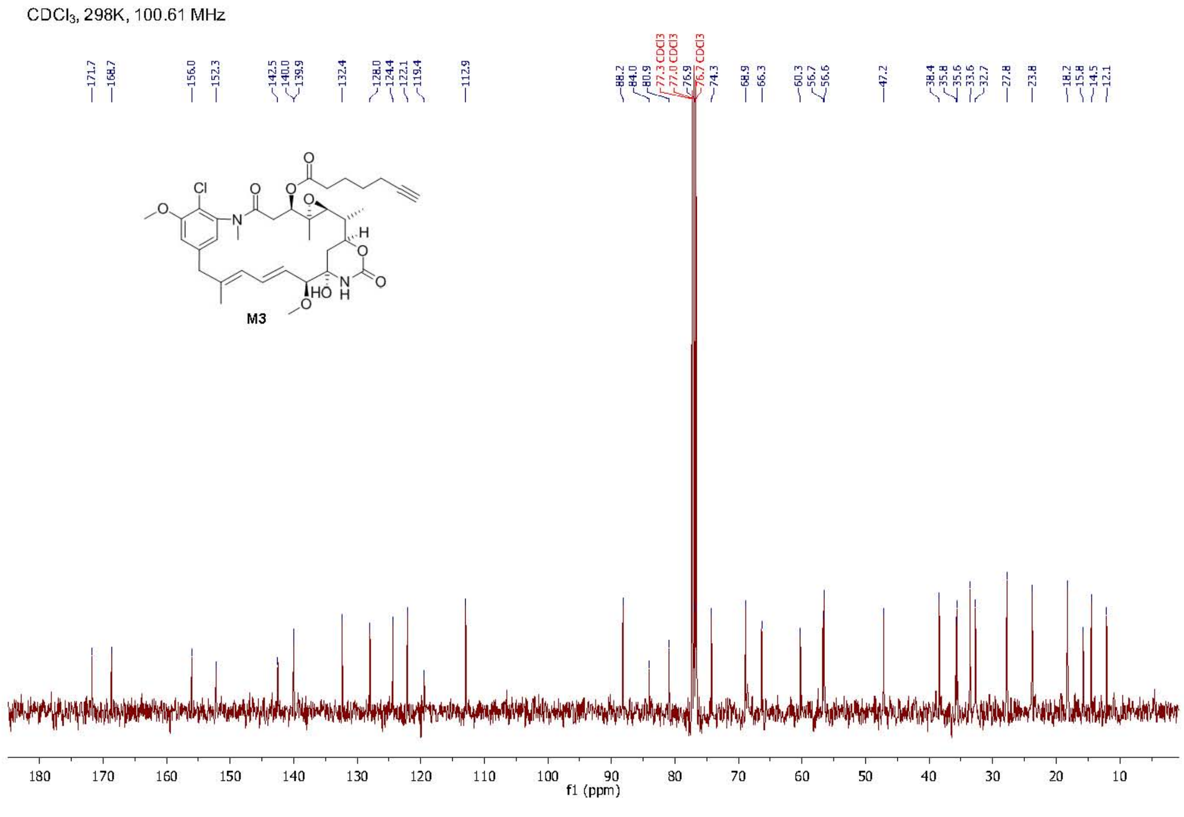

Supplementary Fig. 3: ^{1}H- and ^{13}C-NMR spectra of M3.

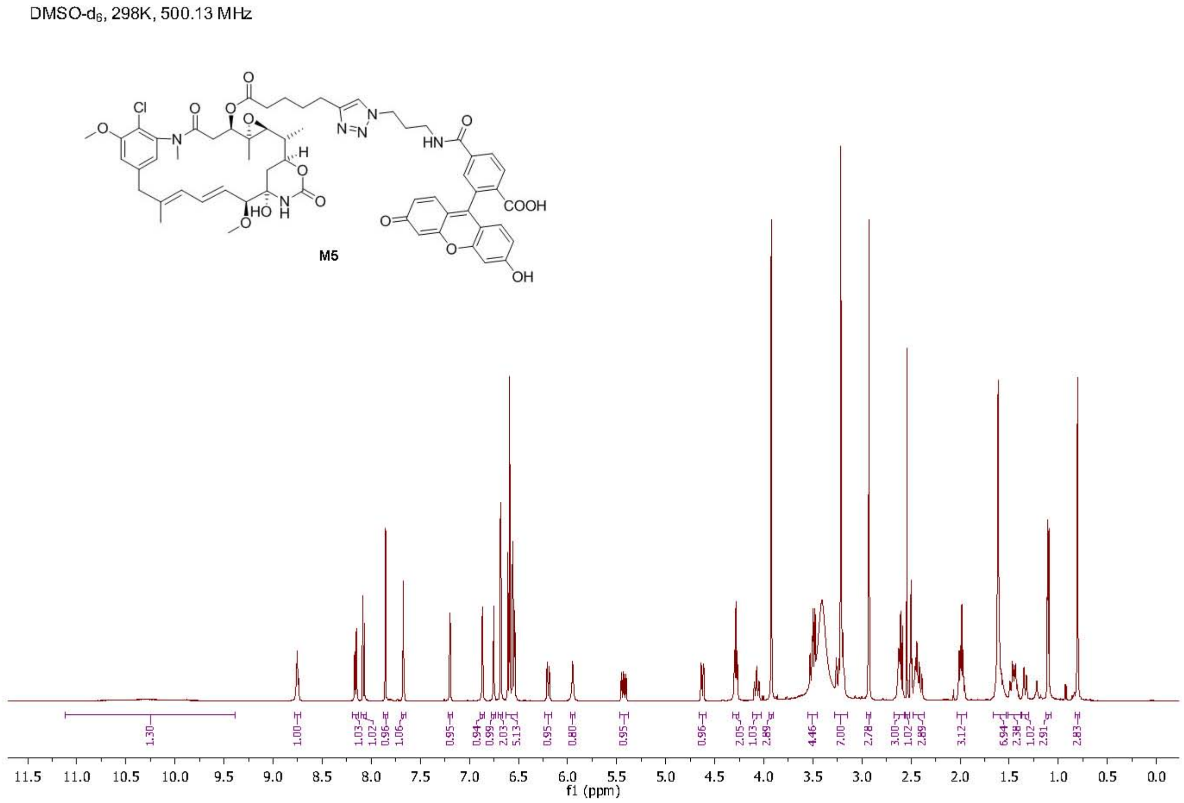

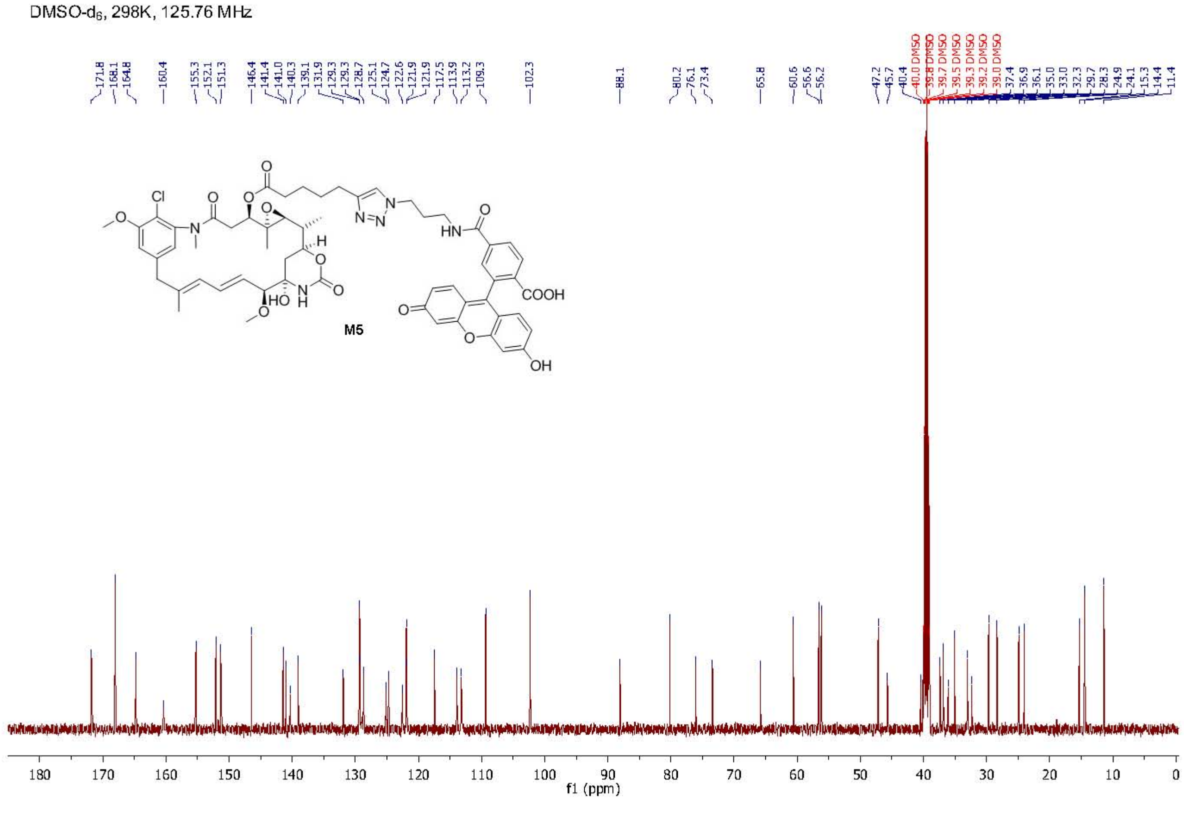

Supplementary Fig. 4: ^{1}H- and ^{13}C-NMR spectra of M5.

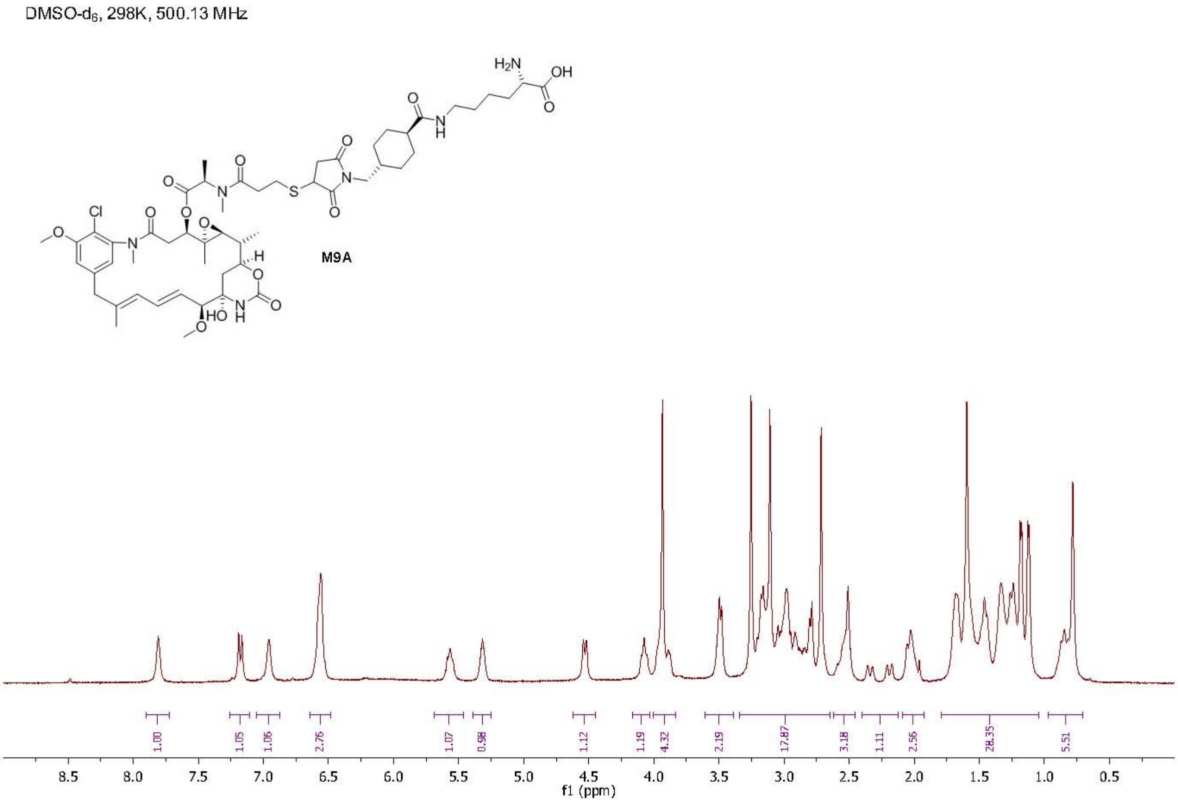

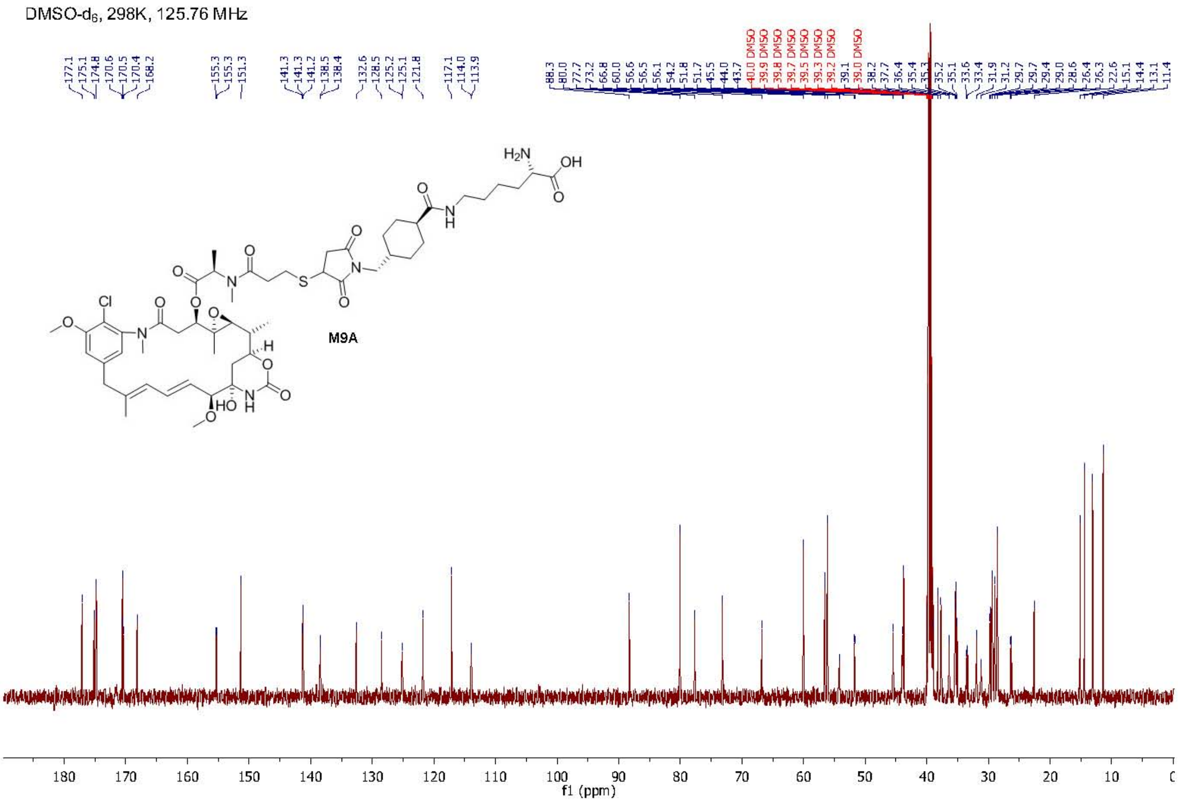

Supplementary Fig. 5: ^{1}H- and ^{13}C-NMR spectra of M9A.

DMSO-d6, 298K, 500.13 MHz

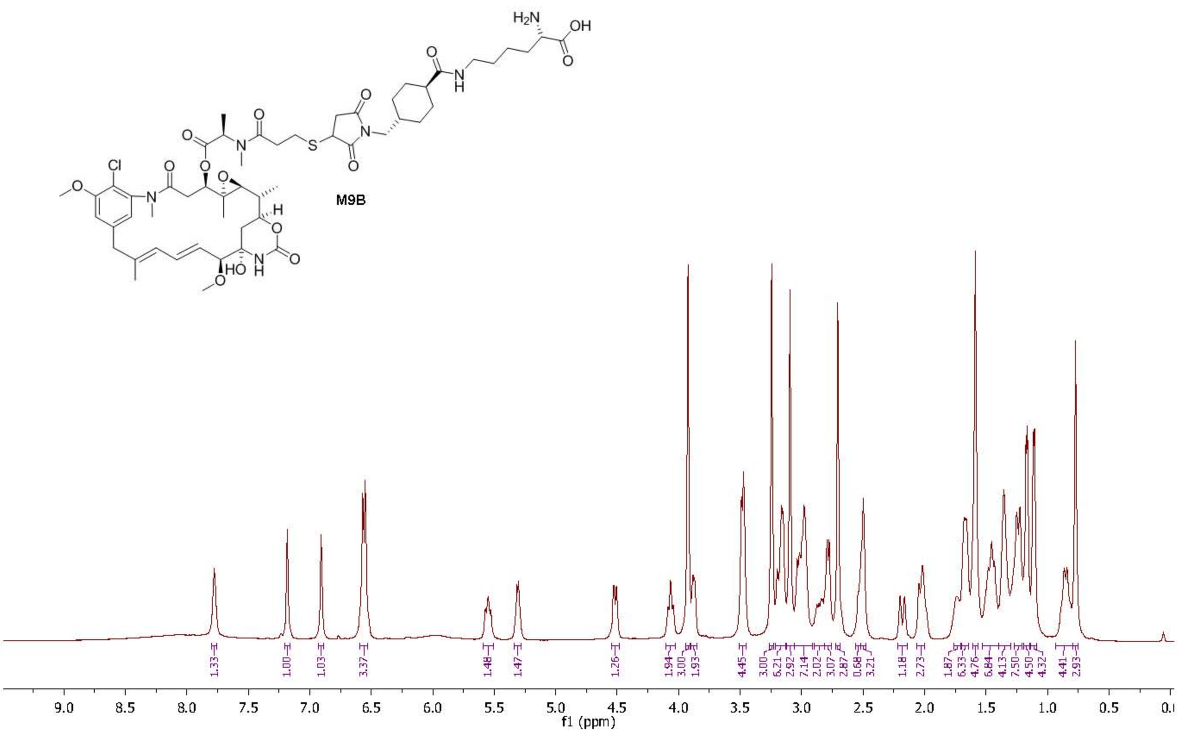

DMSO-d6, 298K, 125.76 MHz

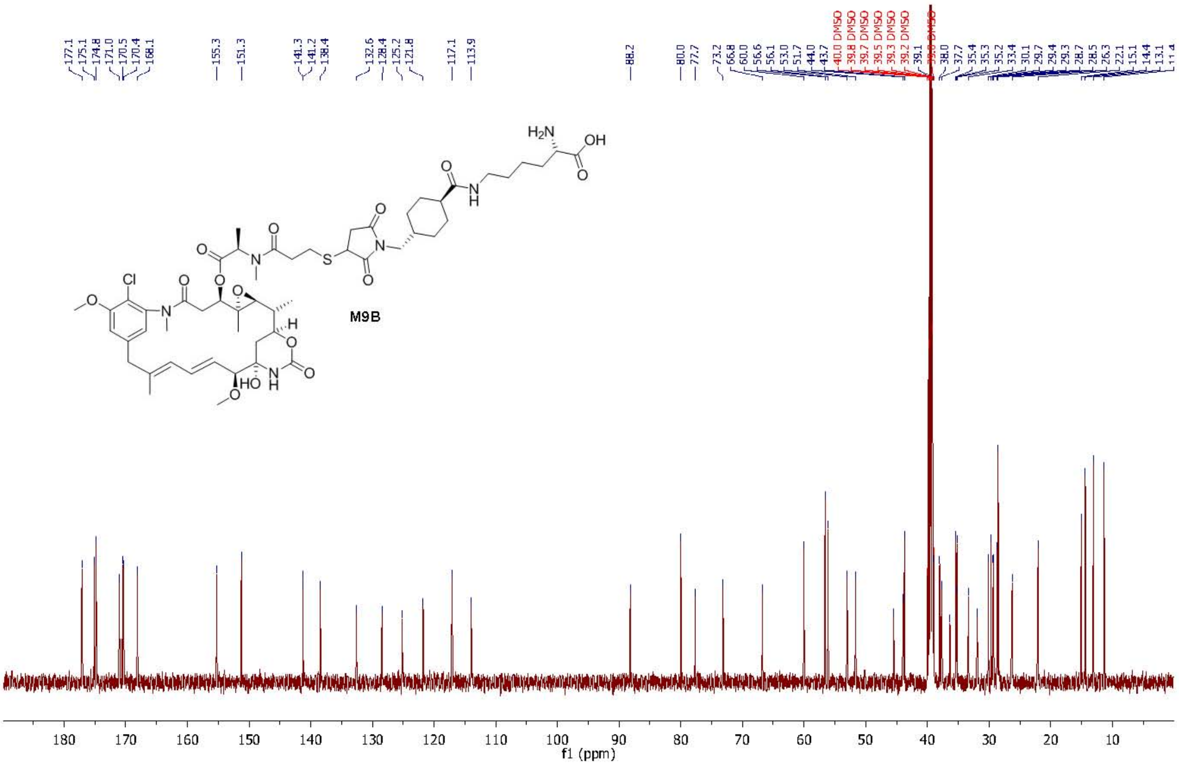

Supplementary Fig. 6: ^{1}H- and ^{13}C-NMR spectra of M9B.

Supplementary Methods

Synthetic Protocols

Maytansinol (**M1**) was purchased from Levena Biopharm, San Diego, USA; DM1 (mertansine, **M6**) was from MCE MedChemExpress. Analytical RP-HPLC was performed on a Hitachi EliteChrom system equipped with a diode array detector, using a Waters Symmetry C18 column (3.5 μM, 4.6x100 mm) at a flow rate of 1 mL/min and UV detection at 254 nm. Preparative RP-HPLC was carried out on a Gilson system using a Waters Symmetry column (5 μM, 19x100 mm) at a flow rate of 15 mL/min and dual UV detection at 254 nm / 211 nm. Eluent solvents were acetonitrile (ACN) and water.

^{1}H and ^{13}C NMR spectra were recorded in $CDCl_3$ or DMSO-d_6 on Bruker AV-400 400 MHz and AV-500 500 MHz instruments at room temperature. Chemical shifts (δ) are reported in ppm and are referenced to the solvent signal as an internal standard ($CDCl_3$ δ=7.26 ppm for ^{1}H, δ=77.00 ppm for ^{13}C). All ^{13}C NMR spectra were measured with complete proton decoupling. Data for NMR spectra are reported as follows: s=singlet, d=doublet, t=triplet, m=multiplet, br=broad signal, *J*=coupling constant in Hz.

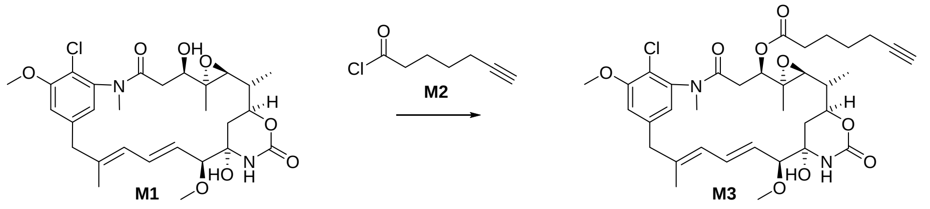

Ester M3: Maytansinol (**M1**) (29 mg, 51 μmol, 1.0 eq) was lyophilized from 1,4-dioxane overnight and then dissolved in CH_2Cl_2 (0.5 mL). To the solution was added Et_3N (70 μL, 0.5 mmol, 10 eq) followed by 4-(1-pyrrolidinyl)pyridine (3.8 mg, 25.6 μmol, 0.5 eq) and hept-6-ynoyl chloride (**M2**) (30 μl, 0.2 μmol, 4.0 eq) (prepared by heating hept-6-ynoic acid with a 7-fold excess of oxalyl chloride in dry CH_2Cl_2 to 70 °C for 1.5 h). The mixture was stirred at rt for 5 d, at which point additional Et_3N (70 μL, 0.5 mmol, 10 eq), 4-(1-pyrrolidinyl)pyridine (3.8 mg, 30 μmol, 0.5 eq) and **M2** (30 μl, 0.2 mmol, 4.0 eq) were supplied; stirring was then continued for another two days. The reaction mixture was directly purified by preparative RP-HPLC (isocratic at 30% ACN for 1 min, then to 70% ACN in 7 min, then isocratic at 70% ACN for 5.5 min) to provide 6.8 mg of ester **M3** (20 %). The ^{1}H-NMR spectrum of this material indicated a significant excess of protons in the region between 2 and 0.5 ppm, which is unaccounted for. The corresponding impurities were not visible in the ^{13}C spectrum, which

gave the expected number of carbons. The material was thus used in the next step without further purification.

^{1}H NMR (400 MHz, $CDCl_3$) δ 6.83 (d, *J* = 1.9 Hz, 1H), 6.78 (d, *J* = 1.9 Hz, 1H), 6.44 (dd, *J* = 15.5, 10.9 Hz, 1H), 6.30 (s, 1H), 6.16 (d, *J* = 11.0 Hz, 1H), 5.49 (dd, *J* = 15.4, 8.9 Hz, 1H), 4.89 (dd, *J* = 12.0, 2.9 Hz, 1H), 4.30 – 4.20 (m, 1H), 3.99 (s, 4H), 3.83 – 3.72 (m, 1H), 3.56 – 3.48 (m, 3H), 3.36 (s, 3H), 3.21 (d, *J* = 28.3 Hz, 6H), 2.89 (d, *J* = 9.8 Hz, 1H), 2.70 (d, *J* = 2.7 Hz, 1H), 2.58 – 2.31 (m, 6H), 2.30 – 2.13 (m, 7H), 2.12 – 2.04 (m, 2H), 1.99 – 0.94 (m, 38H), 0.83 (s, 3H). **^{13}C NMR** (101 MHz, $CDCl_3$) δ 171.7, 168.7, 156.0, 152.3, 142.5, 140.0, 139.9, 132.4, 128.0, 124.4, 122.1, 119.4, 112.9, 88.2, 84.0, 80.9, 76.9, 74.3, 68.9, 66.3, 60.3, 56.7, 56.6, 47.2, 38.4, 35.8, 35.6, 33.6, 32.7, 27.8, 23.8, 18.2, 15.8, 14.5, 12.1. **HRMS** (ESI): *m/z* calcd for $C_{35}H_{46}ClN_2O_9$ 673.2886 $[M+H]^+$; found 673.2887.

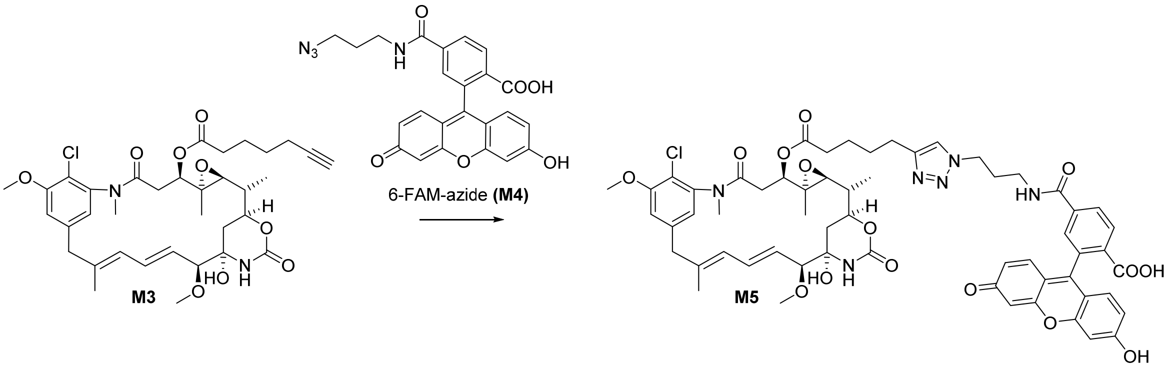

Triazole M5: To a solution of ester **M3** (6.8 mg, 10 µmol, 1.0 eq) and 6-FAM-azide (**M4**) (4.6 mg, 10 µmol, 1.0 eq) in 0.4 mL THF:H_2O (4:1) was added $CuSO_4$ (0.3 mg, 1 µmol, 0.1 eq) and the mixture was purged with a stream of Ar for 10 min. An aqu. solution of (+)-sodium-L-ascorbate (0.8 mg, 4 µmol, 0.4 eq, in 34 µL H_2O; an aliquot of a stock solution that had been purged with Ar) was then added under Ar at rt. The mixture was stirred for 3 h at rt, at which point HPLC analysis showed full consumption of 6-FAM-azide (**M4**), while ca. 50% of **M3** were still present. Further 6-FAM-azide (2.3 mg, 5 µmol, 0.5 eq) was thus added and stirring was continued for another 2 h. After concentration and dilution into 1 mL DMSO, the mixture was directly purified by preparative RP-HPLC (isocratic at 30% ACN for 1 min, then to 70% ACN in 10 min) to furnish **M5** (3.25 mg, 2.9 µmol, 29 %) as a yellow solid. 1.9 mg of ester **M3** (2.8 µmol, 28 %) could be reisolated.

^{1}H NMR (500 MHz, DMSO-d_6) δ 10.29 (s, br, 1H), 8.75 (t, *J* = 5.6 Hz, 1H), 8.16 (dd, *J* = 8.1, 1.4 Hz, 1H), 8.08 (d, *J* = 8.0 Hz, 1H), 7.85 (s, 1H), 7.70 – 7.65 (m, 1H), 7.20 (d, *J* = 1.8 Hz, 1H), 6.87 (s, 1H), 6.75 (d, *J* = 1.7 Hz, 1H), 6.68 (d, *J* = 2.3 Hz, 2H), 6.63 – 6.51 (m, 5H), 6.23 – 6.16 (m, 1H), 5.95 (s, 1H), 5.43 (dd, *J* = 15.3, 8.9 Hz, 1H), 4.63 (dd, *J* = 11.9, 2.7 Hz, 1H),

4.28 (t, J = 7.0 Hz, 2H), 4.07 (ddd, J = 12.2, 10.4, 2.1 Hz, 1H), 3.92 (s, 3H), 3.55 – 3.46 (m, 4H), 3.28 – 3.15 (m, 7H), 2.93 (s, 3H), 2.61 (dd, J = 12.8, 6.6 Hz, 3H), 2.54 (s, 1H), 2.48 – 2.37 (m, 3H), 1.99 (dt, J = 14.0, 4.6 Hz, 3H), 1.66 – 1.53 (m, 7H), 1.45 (tdd, J = 12.8, 10.8, 5.1 Hz, 2H), 1.34 (d, J = 13.1 Hz, 1H), 1.10 (d, J = 6.4 Hz, 3H), 0.80 (s, 3H). **^{13}C NMR** (126 MHz, DMSO) δ 171.8, 168.1, 164.8, 160.4, 155.3, 152.1, 151.3, 146.4, 141.4, 141.0, 140.3, 139.1, 131.9, 129.3, 129.3, 128.7, 125.1, 124.7, 122.6, 121.9, 121.9, 117.5, 113.9, 113.2, 109.3, 102.3, 88.1, 80.2, 76.1, 73.4, 65.8, 60.6, 56.6, 56.2, 47.2, 45.7, 40.4, 37.4, 36.9, 36.1, 35.0, 33.0, 32.3, 29.7, 28.3, 24.9, 24.1, 15.3, 14.4, 11.4. **HRMS** (ESI): *m/z* calcd for $C_{59}H_{64}ClN_6O_{15}$ 1131.4113 [M+H]$^+$; found 1131.4099. **RP-HPLC**: (t_R 6.87 min, 30% ACN to 70% ACN over 10 min).

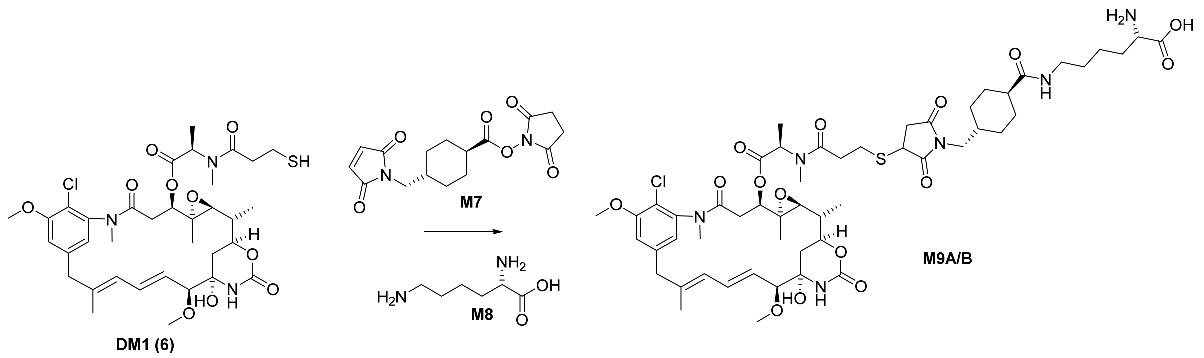

Lys-conjugate M9: DM1 (**M6**) (21.7 mg, 29 µmol, 1.0 eq) and L-lysine (**M8**) (47 mg, 0.32 mmol, 11.0 eq) were suspended in a mixture of 0.9 mL phosphate buffer (pH 7.2) and 1.8 mL dimethylacetamide. After two minutes, **M7** (10 mg, 32 µmol, 1.1 eq) was added as a solution in 0.2 mL dimethyl acetamide (DMA) at rt. The reaction mixture was stirred for 1.5 h at rt and then filtered and the residue washed with 1 ml DMA. After concentration in high vacuum the residue was redissolved in H_2O:DMSO (1:1) and the solution was directly submitted to preparative RP-HPLC (1 min isocractic at 10% CAN, then to 60% ACN over 15 min). After a first round of purification (in three individual injections), each of the two C9'-diastereoisomers formed in the reaction was obtained with *ca*. 80% HPLC purity. The slower eluting isomer (**M9B**) was repurified once under the same conditions to provide 2.26 mg of material of *ca*. 95% HPLC purity (t_R 14.63 min, 10% ACN to 50% ACN over 15 min). For the faster eluting isomer (**M9A**) two more rounds of purification under the same conditions furnished 3.18 mg of material of 95% HPLC purity (t_R 14.36 min, 10% ACN to 50% ACN over 15 min).

M9A: ^{1}H NMR (500 MHz, DMSO-d_6) δ 7.81 (d, J = 5.7 Hz, 1H), 7.18 (d, J = 12.7 Hz, 1H), 7.05 – 6.87 (m, 1H), 6.64 – 6.48 (m, 3H), 5.69 – 5.46 (m, 1H), 5.39 – 5.25 (m, 1H), 4.53 (d, J = 11.9 Hz, 1H), 4.07 (t, J = 11.1 Hz, 1H), 4.00 – 3.83 (m, 4H), 3.50 (t, J = 8.0 Hz, 2H), 3.34 – 2.65 (m, 18H), 2.53 (d, J = 21.9 Hz, 3H), 2.26 (dd, J = 74.5, 18.2 Hz, 1H), 2.09 – 1.92 (m, 3H), 1.79 – 1.05 (m, 28H), 0.97 – 0.70 (m, 6H). **^{13}C NMR** (126 MHz, DMSO) δ 177.1, 175.1, 174.8, 170.6, 170.5, 170.4, 168.2, 155.3, 155.3, 151.3, 141.3, 141.3, 141.2, 138.5, 138.4, 132.6, 128.5, 125.2, 125.1, 121.8, 117.1, 114.0, 113.9, 88.3, 80.0, 77.7, 73.2, 66.8, 60.0, 56.6, 56.5, 56.1, 54.2, 51.8, 51.7, 45.5, 44.0, 43.7, 39.1, 38.2, 37.7, 36.4, 35.4, 35.3, 35.2, 35.1, 33.6, 33.4, 31.9, 31.2, 29.7, 29.7, 29.4, 29.0, 28.6, 26.4, 26.3, 22.6, 15.1, 14.4, 13.1, 11.4.

M9B: ^{1}H NMR (500 MHz, DMSO-d_6) δ 7.78 (s, 1H), 7.18 (s, 1H), 6.91 (s, 1H), 6.56 (d, *J* = 10.3 Hz, 3H), 5.59 – 5.51 (m, 1H), 5.31 (d, *J* = 6.3 Hz, 1H), 4.52 (d, *J* = 11.5 Hz, 1H), 4.07 (t, *J* = 10.6 Hz, 2H), 3.92 (s, 3H), 3.90 – 3.85 (m, 2H), 3.48 (d, *J* = 8.4 Hz, 4H), 3.24 (s, 3H), 3.21 – 3.13 (m, 6H), 3.10 (s, 3H), 3.01 (dd, *J* = 19.5, 8.6 Hz, 7H), 2.85 (dd, *J* = 15.6, 6.3 Hz, 2H), 2.78 (d, *J* = 8.8 Hz, 3H), 2.71 (s, 3H), 2.54 (s, 1H), 2.50 (s, 3H), 2.18 (d, *J* = 18.1 Hz, 1H), 2.03 (d, *J* = 14.3 Hz, 3H), 1.76 – 1.71 (m, 2H), 1.70 – 1.64 (m, 6H), 1.59 (s, 5H), 1.53 – 1.40 (m, 7H), 1.35 (s, 4H), 1.24 (d, *J* = 12.5 Hz, 8H), 1.17 (d, *J* = 6.0 Hz, 5H), 1.11 (d, *J* = 5.3 Hz, 4H), 0.93 – 0.80 (m, 4H), 0.77 (s, 3H).

^{13}C NMR (126 MHz, DMSO) δ 177.1, 175.1, 174.8, 171.0, 170.5, 170.4, 168.1, 155.3, 151.3, 141.3, 141.2, 138.4, 132.6, 128.4, 125.2, 121.8, 117.1, 113.9, 88.2, 80.0, 77.7, 73.2, 66.8, 60.0, 56.6, 56.1, 53.0, 51.7, 45.5, 44.0, 43.7, 39.1, 38.0, 37.7, 36.3, 35.4, 35.3, 35.2, 33.4, 31.9, 30.1, 29.7, 29.4, 29.3, 28.7, 28.5, 26.3, 22.1, 15.1, 14.4, 13.1, 11.4.

HRMS (ESI) **(Mixture M9A/B)**: *m/z* calcd for $C_{53}H_{76}ClN_6O_{15}S$ 1103.4772 $[M+H]^+$; found 1103.4769. (Low resolution) HPLC-MS of the mixture showed an identical mass for both peaks (1103.0 in both cases ($[M+H]^+$).

Supplementary References

1. Karplus, P.A. & Diederichs, K., Linking crystallographic model and data quality, Science **336**, 1030-1033 (2012).

2. Davis, I.W., Murray, L.W., Richardson, J.S., & Richardson, D.C., MOLPROBITY: structure validation and all-atom contact analysis for nucleic acids and their complexes, Nucleic Acids Res. **32**, W615-W619 (2004).

3. Zhang, J.H., Chung, T.D., & Oldenburg, K.R., A Simple Statistical Parameter for Use in Evaluation and Validation of High Throughput Screening Assays, J. Biomol. Screen. **4**, 67-73 (1999).
